# Use of lipid parameters to identify apparently healthy men at high risk of arterial stiffness progression

**DOI:** 10.1186/s12872-020-01846-x

**Published:** 2021-01-13

**Authors:** Yu Sang, Ming Cao, Xiaofen Wu, Lei Ruan, Cuntai Zhang

**Affiliations:** Department of Geriatrics, Tongji Hospital of Tongji Medical College, Huazhong University of Science and Technology, Wuhan, China

**Keywords:** Lipids, Triglyceride, TG/HDL-C, Arterial stiffness, Pulse wave velocity

## Abstract

**Background:**

Dyslipidemia contributes to the development and progression of arterial stiffness. We aimed to identify the most informative measures of serum lipids and their calculated ratios in terms of arterial stiffness progression risk.

**Methods:**

Total cholesterol (TC), triglyceride (TG), low-density lipoprotein cholesterol (LDL-C), high-density lipoprotein cholesterol (HDL-C), and brachial-ankle pulse wave velocity (baPWV) of 659 healthy males (47.4 ± 10.7 years) were measured at baseline. Values for non-HDL-C, TC/HDL-C, TG/HDL-C, LDL-C/HDL-C, and non-HDL-C/HDL-C were calculated. BaPWV was re-performed after 4.1 years follow-up. Elevated baPWV was defined as baPWV ≥ 1400 cm/s.

**Results:**

Over the follow-up period, the mean baPWV value increased from 1340 cm/s to 1410 cm/s, and 331 individuals increased/persisted with high baPWV (outcome 1). Among the 448 subjects who had normal baseline baPWV, 100 incident elevated baPWV occurred (outcome 2). Only baseline logTG (OR 1.64 [95% CI: 1.14–2.37] for outcome 1; 1.89 [1.14–3.17] for outcome 2) and logTG/HDL-C (1.54 [1.15–2.10] for outcome 1; 1.60 [1.05–2.45] for outcome 2) were significantly associated with arterial stiffness progression after adjusting for confounding factors. Adding logTG or logTG/HDL-C to age and blood pressure improved the accuracy of risk predictions for arterial stiffness progression. These associations remained significant when lipids were analyzed as categorical variables.

**Conclusions:**

Baseline serum TG and TG/HDL-C were independently associated with increases in/persistently high baPWV and incident elevated baPWV, and they performed more effectively than other lipid variables in identifying healthy men at high risk of arterial stiffness progression.

## Background

Cardiovascular disease (CVD) has become the leading cause of death in China [1]. The increase in CVD risk is largely driven by adverse changes of the vasculature, including arterial stiffening. Brachial-ankle pulse wave velocity (baPWV) is the most widely used routine clinical practice in Asia to assess arterial stiffness and is being increasingly incorporated into studies in the US and Europe [2, 3], since the measurement is valid, reproducible, minimal-risk, convenient, and cost-saving. Accumulating evidence demonstrates that arterial stiffness, evaluated by baPWV, is an independent predictor for CVD events and mortality in the general population and various patient populations [4–6]. Arterial stiffness is also one of the major age-related arterial phenotypes [7], and the ‘Vascular Aging Continuum’ has regarded increased arterial stiffness as the fundamental and vital link [8]. Pulse wave velocity is considered a physiological method for quantifying vascular aging [9, 10]. Therefore, a relatively simple means to identify individuals at high risk of arterial stiffness progression would be clinically useful.

Dyslipidemia characterized by the increase of total cholesterol (TC), triglyceride (TG), or low-density lipoprotein cholesterol (LDL-C), or the decrease of high-density lipoprotein cholesterol (HDL-C) contributes to arterial stiffness and has been proven to be associated with baPWV in extensive cross-sectional studies [7, 11]. However, associations observed in cross-sectional analyses limit inferences about temporality and longitudinal studies regarding the role of serum lipids on arterial stiffness progression are limited and far from conclusive. In addition, arterial stiffness progresses at different rates and can be accelerated by several long-standing cardiovascular risk factors [12], and participants enrolled in previous longitudinal studies were often affected by a variety of chronic diseases. Therefore we selected apparently healthy individuals to reduce the potential bias from other diseases and capture the dyslipidemia-related atrial stiffness progression difference. We aimed to determine the association of baPWV progression with baseline serum lipids, including TC, TG, LDL-C, HDL-C, non-HDL-C, TC/HDL-C, TG/HDL-C, LDL-C/HDL-C, and non-HDL-C/HDL-C, and to confirm the most informative one of those lipid parameters in identifying healthy men at high risk of arterial stiffness progression.

## Methods

### Subjects

**Fig. 1:**
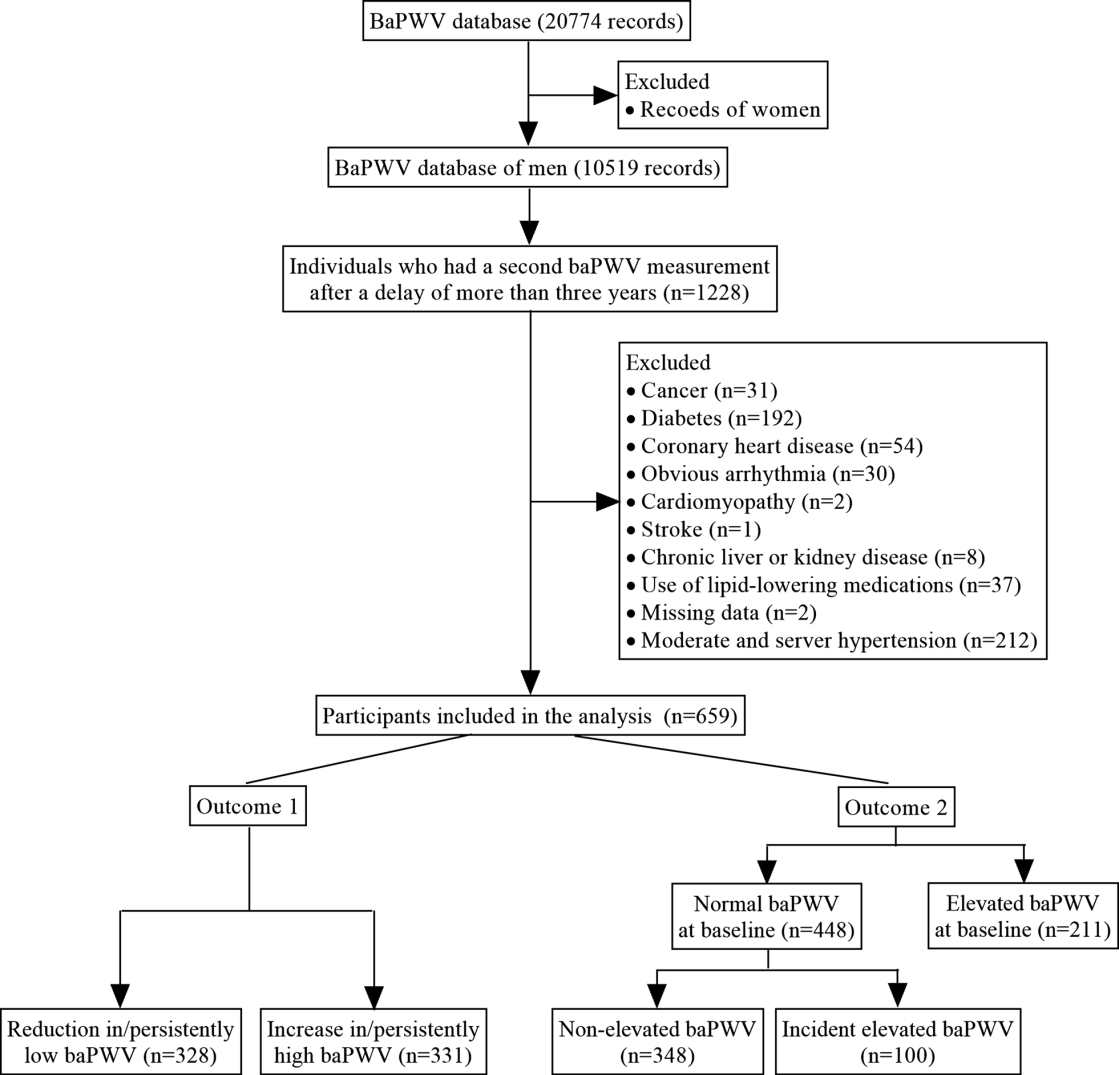
Flowchart describing the selection and categorization of participants in the study. BaPWV indicates brachial-ankle pulse wave velocity

This retrospective longitudinal study was conducted at the physical examination center of the geriatric department of Tongji Hospital. The center is not confined to the elderly and is mainly responsible for employee health screening of local enterprises. The study was approved by the medical ethics committee of Tongji Hospital (TJ-IRB20190410) and the study protocol conforms to the Declaration of Helsinki. Considering the small sample size of women in our center and the sex difference in arterial stiffness [13], we screened the 10,519 baPWV database records of males from March 2011 to July 2019. A total of 1228 individuals had their second baPWV measurement after a delay of more than three years. The exclusion criteria were: age < 18 years, inheritable dyslipidemia, use of lipid-lowering medications, moderate or server hypertension [defined as systolic blood pressure (SBP) ≥ 150 mm Hg, diastolic blood pressure (DBP) ≥ 95 mm Hg, or use of antihypertensive drugs], diabetes [defined as fasting blood glucose (FBG) of ≥ 7.0 mmol/L, HbA1c ≥ 6.5%, or use of diabetes medications], coronary heart disease, stroke, obvious arrhythmia (persistent atrial fibrillation, frequent premature beats, or wearing a pacemaker), cardiomyopathy, valvular heart disease, chronic liver or kidney disease, cancer, ankle-brachial index (ABI) less than 0.9 [14], and missing data. Finally, 659 subjects were included in the analysis (Fig. 1).

### Baseline clinical characteristics

Standardized in-person interviews were conducted by trained staff to collect information regarding age, sex, current cigarette smoking status, medical history, and medication use. Anthropometric indexes including height and weight were measured. Body mass index (BMI) was computed as the weight in kilograms divided by the square of the height in meters. Blood pressure and heart rate were measured using an OMRON sphygmomanometer (OMRON Corporation, Japan). The blood pressure and heart rate used in the analysis were calculated as the average of three measured values. Mean arterial pressure (MAP) was calculated from the standard equation MAP = (2/3)DBP + (1/3)SBP (in mm Hg). Fasting venous blood samples were collected and sent to the hospital’s clinical chemistry laboratory. TC, TG, LDL-C, HDL-C, FBG, HbA1c, creatinine, and uric acid were measured using standard certified assays. We also calculated values for non-HDL-C (TC minus HDL-C), TC/HDL-C (TC divided by HDL-C), TG/HDL-C (TG divided by HDL-C), LDL-C/HDL-C (LDL-C divided by HDL-C), and non-HDL-C/HDL-C (non-HDL-C divided by HDL-C).

### Arterial stiffness measurements at baseline and follow-up and definition of the outcomes

BaPWV and ABI were measured using the Vascular Profiler BP-203RPEIII system (Omron, Kyoto, Japan). Trained technicians placed the pressure cuffs on the subjects, i.e., one on the upper part of each arm and one on each ankle. Then, the subjects were examined after ten minutes of rest in the supine position. The device simultaneously recorded the bilateral pulse waves of the brachial and posterior tibial arteries using an oscillometric method. BaPWV was calculated as the ratio of the traveled distance (which was automatically estimated from the body height) divided by the transit time of the pulse wave between the brachial and posterior tibial arteries. We classified the outcome in two different manners. Outcome 1: the baseline and follow-up baPWV were both divided into quartiles, respectively. Then we classified subjects into two subgroups: those who decreased their quartile distribution or persisted within the two lower quartile groups, and those who increased their quartile distribution or persisted within the two higher quartile groups [15]. Outcome 2: a cutoff value of more than 1400 cm/s for baPWV was used to diagnose elevated baPWV [4, 16–18]. Individuals with normal baseline baPWV were divided into non-elevated baPWV and incident elevated baPWV groups based on their follow-up baPWV levels.

### Statistical analysis

Data were analyzed using R and RStudio 3.6.2. Continuous variables were presented as the means ± standard deviation or medians (interquartile range), as appropriate for the distribution. Categorical variables were shown as counts and proportions. Paired t-tests were used to determine if the baPWV levels changed over the follow-up period. We compared the baseline variables between groups using unpaired t-test, Mann–Whitney U test, and Chi-squared test accordingly. For comparison, identical crude and multivariable-adjusted binary logistic regression models were built for each lipid parameter respectively. Odds ratios (ORs) and 95% confidence intervals (CIs) for the two outcomes were calculated. Prior to regression analysis, TG and TG/HDL-C were log-transformed to achieve normality. Age and MAP, the most important determinants for baPWV, were controlled for in the first model. Further adjustments were made for BMI, current smoking status, heart rate, FBG, creatinine, and uric acid in the second model. All the covariates included were measured at baseline. The improvement in the ability of each serum lipid to predict outcomes was summarized using area under the receiver operating characteristic (ROC) curves (AUC), positive net reclassification improvement (NRI), and integrated discrimination improvement (IDI) [19]. In sensitivity analyses, TC, TG, HDL-C, LDL-C, and non-HDL were divided into three or two levels by clinical cut-points [20]; TC/HDL-C, TG/HDL-C, LDL-C/HDL-C, and non-HDL-C/HDL-C ratios were divided into three levels by tertile cut-points. ORs and 95% CIs for the outcomes across categories of each serum lipid were also calculated, using the most favorable category as the reference. Trends in ORs across categories of each lipid were calculated by modeling the lipid categories as an ordinal variable. Two-tailed *p*-values < 0.05 were considered significant.

## Results

### General characteristics

The clinical characteristics of the study subjects are shown in Table 1. The population had a mean baseline age of 47.4 ± 10.7 years, ranging from 22 to 94 years. Sixty-seven (10.2%) individuals were aged 60 years or older, and 6 of them were the oldest-old (aged 80 and older). The median duration of the follow-up was 4.4 years. The mean baPWV value increased from 1340 ± 209 cm/s to 1410 ± 245 cm/s over the follow-up period (*p* < 0.001).

**Table 1:** Baseline characteristics of the individuals included in the analysis

Variables	Total (n = 659)	Reduction in/persistently low baPWV (n = 328)	Increase in/persistently high baPWV (n = 331)	*p* value^a^	Non-elevated baPWV (n = 348)	Incident elevated baPWV (n = 100)	*p* value^b^
Age, years	47.4 ± 10.7	43.4 ± 8.7	51.5 ± 10.9	< 0.001	43.2 ± 8.8	49.8 ± 7.9	< 0.001
Smoker, %	241 (36.6)	125 (38.1)	116 (35.0)	0.46	140 (40.2)	34 (34.0)	0.31
BMI, kg/m2	24.6 ± 2.8	24.7 ± 2.6	24.6 ± 2.9	0.84	24.6 ± 2.8	24.5 ± 3.1	0.71
SBP, mm Hg	121 ± 11	119 ± 11	123 ± 11	< 0.001	117 ± 10	121 ± 10	0.003
DBP, mm Hg	75 ± 9	73 ± 9	76 ± 9	< 0.001	71 ± 8	75 ± 8	< 0.001
MAP, mm Hg	90 ± 9	89 ± 9	92 ± 9	< 0.001	87 ± 8	90 ± 8	< 0.001
Heart rate, beats/min	67 ± 10	66 ± 10	68 ± 9	0.008	65 ± 9	66 ± 8	0.10
ABI	1.10 ± 0.08	1.09 ± 0.08	1.11 ± 0.08	0.005	1.09 ± 0.08	1.11 ± 0.08	0.03
FBG, mmol/L	5.04 ± 0.52	4.99 ± 0.50	5.08 ± 0.54	0.03	4.97 ± 0.50	5.08 ± 0.53	0.06
HbA1c, %	5.58 ± 0.31	5.55 ± 0.31	5.61 ± 0.30	0.02	5.54 ± 0.30	5.61 ± 0.27	0.05
creatinine, μmol/L	81.0 ± 10.8	81.2 ± 10.2	80.8 ± 11.3	0.68	81.3 ± 10.4	80.7 ± 11.9	0.60
Uric acid, umol/L	376 ± 76	376 ± 76	376 ± 76	0.94	373 ± 75	385 ± 80	0.18
TC, mmol/L	4.74 ± 0.86	4.74 ± 0.83	4.74 ± 0.90	0.97	4.71 ± 0.83	4.78 ± 0.92	0.50
TG, mmol/L	1.36 (1.01)	1.30 (0.83)	1.42 (1.09)	0.005	1.29 (0.80)	1.60 (1.08)	0.005
LDL-C, mmol/L	2.94 ± 0.74	2.96 ± 0.69	2.92 ± 0.78	0.43	2.96 ± 0.72	2.91 ± 0.81	0.62
Non-HDL-C, mmol/L	3.56 ± 0.84	3.54 ± 0.81	3.59 ± 0.87	0.52	3.52 ± 0.83	3.61 ± 0.90	0.39
HDL-C, mmol/L	1.18 ± 0.26	1.20 ± 0.25	1.16 ± 0.28	0.06	1.19 ± 0.24	1.17 ± 0.32	0.60
TC/HDL-C	4.18 ± 1.02	4.10 ± 0.98	4.26 ± 1.06	0.04	4.09 ± 1.02	4.26 ± 1.06	0.16
TG/HDL-C	1.21 (1.04)	1.11 (0.87)	1.30 (1.22)	0.003	1.11 (0.90)	1.51 (1.35)	0.01
LDL-C/HDL-C	2.59 ± 0.74	2.56 ± 0.71	2.61 ± 0.78	0.32	2.56 ± 0.73	2.61 ± 0.82	0.65
Non-HDL-C/HDL-C	3.18 ± 1.02	3.10 ± 0.98	3.26 ± 1.06	0.04	3.09 ± 1.02	3.26 ± 1.06	0.15
Baseline baPWV, cm/s	1340 ± 209	1280 ± 165	1410 ± 226	< 0.001	1210 ± 100	1300 ± 70	0.001
Follow-up baPWV, cm/s	1410 ± 245	1250 ± 112	1570 ± 240	< 0.001	1250 ± 95	1520 ± 99	0.001

Data are mean ± standard deviation, median (interquartile range), or n (%)

*BMI *body mass index, *SBP* systolic blood pressure, *DBP* diastolic blood pressure, *MAP* mean arterial pressure, *ABI* ankle-brachial index, *FBG* fasting blood glucose, *TC* total cholesterol, *TG* triglyceride, *LDL-C* low-density lipoprotein cholesterol, *HDL-C* high-density lipoprotein cholesterol

^a^Reduction in/persistently low baPWV versus increase in/persistently high baPWV

^b^Non-eleated baPWV versus incident elevated baPWV. *p* values were calculated using unpaired t-test, Mann–Whitney U test, or χ^2^ test, as appropriate

Of all the study participants, 331 (50.2%) individuals had increased/persisted with high baPWV (outcome 1), and they had higher levels of age, blood pressure, heart rate, ABI, FBG, HbA1c, TG, TC/HDL-C, TG/HDL-C, and non-HDL-C/HDL-C at baseline, compared with those who presented a reduction or persisted with low baPWV. There were no statistical differences in other lipids, including TC, LDL-C, non-HDL-C, HDL-C, and LDL-C/HDL-C.

A total of 448 subjects had normal baseline baPWV, and 100 (22.3%) of them had elevated baPWV at follow-up (outcome 2). Individuals with incident elevated baPWV had higher levels of age, blood pressure, ABI, HbA1c, TG, and TG/HDL-C, compared with those who stayed arterial health. Other lipids, including TC, LDL-C, non-HDL-C, HDL-C, TC/HDL-C, LDL-C/HDL-C, and non-HDL-C/HDL-C had no statistical difference.

### Arterial stiffness progression and baseline lipid parameters as continuous variables

**Fig. 2:**
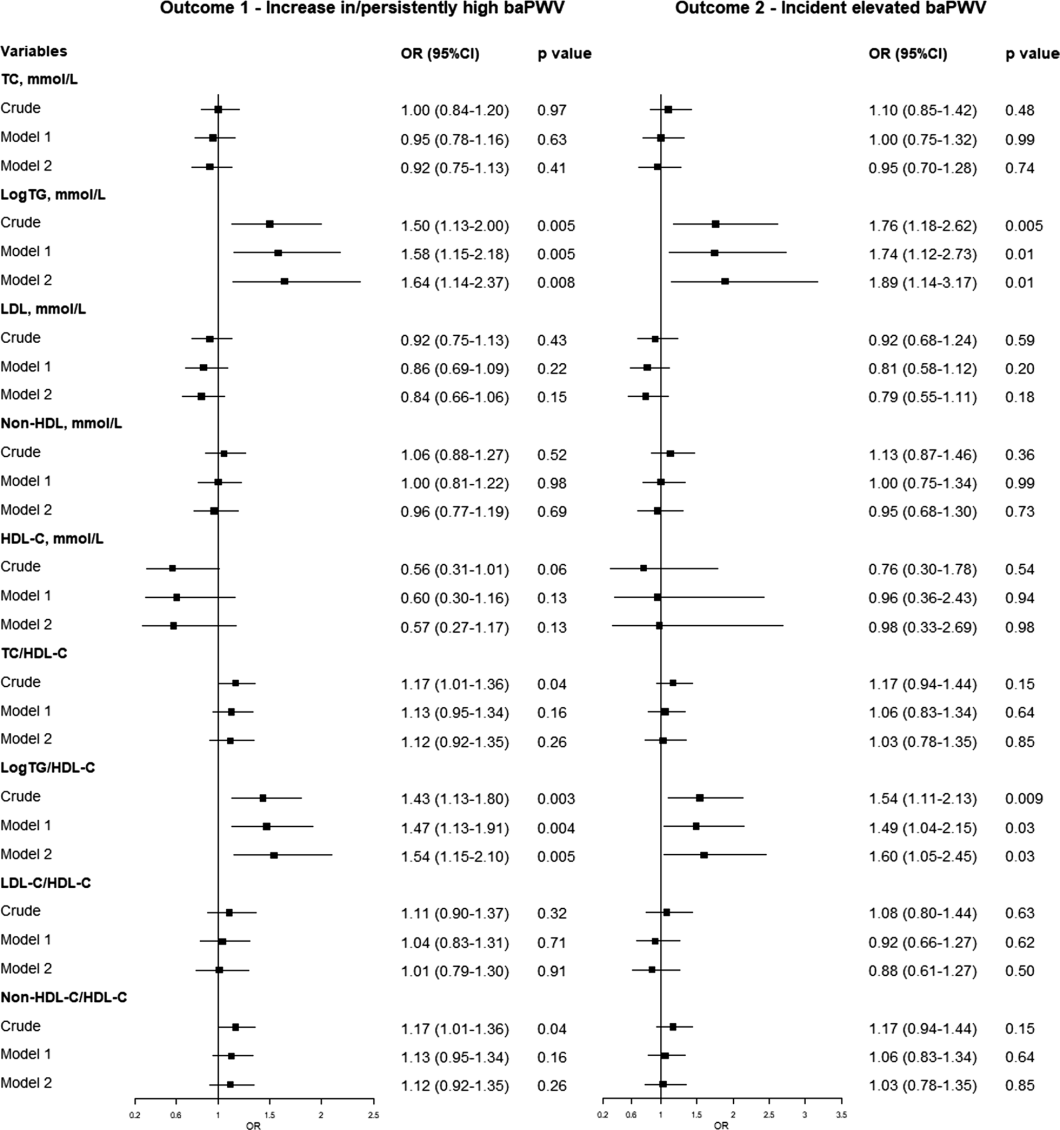
Forest plot of ORs of continuous lipids for arterial stiffness progression. Left: outcome 1 (increases in/persistently high baPWV). Right: outcome 2 (incident elevated baPWV). Model 1: adjustmet for baseline age and mean arterial pressure. Model 2: adjustment for baseline age, mean arterial pressure, body mass index, smoking, heart rate, fasting blood glucose, creatinine, and uric acid. BaPWV indicates brachial-ankle pulse wave veloctiy; TC, total cholesterol; TG, triglyceride; LDL-C, low-density lipoprotein cholesterol; HDL-C, high-density lipoprotein cholesterol

Only baseline logTG and logTG/HDL-C were significantly associated with increased risk of arterial stiffness progression in both crude and adjusted logistic regression models (all *p* < 0.05; Fig. 2). After adjusting for the baseline confounding factors, a unit increase in baseline logTG and logTG/HDL-C resulted in ORs for outcome 1 of 1.64 (95% CI: 1.14–2.37) and 1.54 (95% CI: 1.15–2.10), respectively. Meanwhile, the ORs for outcome 2 per unit increase in baseline logTG and logTG/HDL-C was 1.89 (95% CI: 1.14–3.17) and 1.60 (95% CI: 1.05–2.45), respectively. TC/HDL-C and non-HDL-C/HDL-C were not significantly associated with arterial stiffness progression in the adjusted models, while the ORs of TC, LDL-C, non-HDL-C, HDL-C, and LDL-C/HDL-C did not reach statistical significance in all the models.

**Fig. 3:**
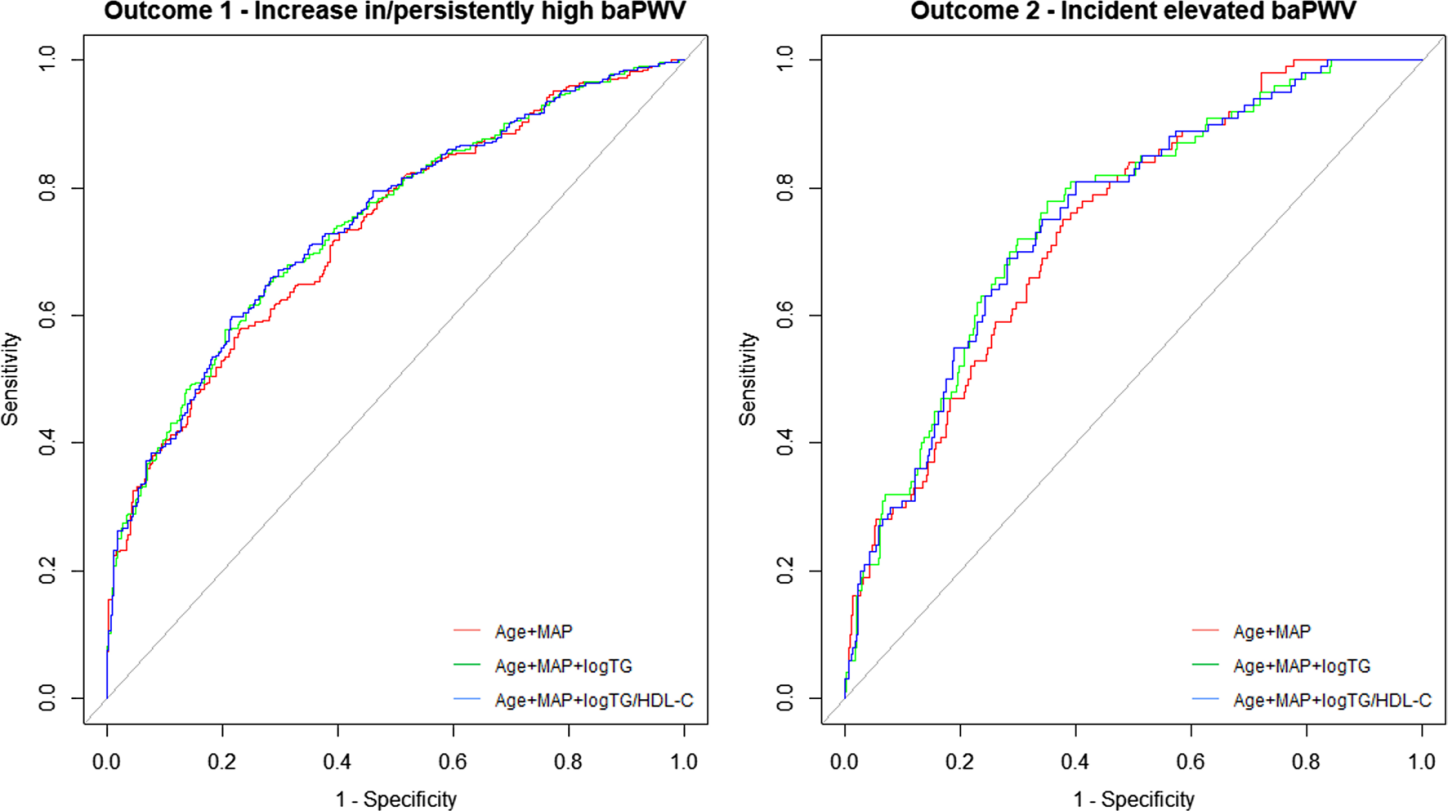
ROC analyses of logTG and logTG/HDL-C for the prediction of arterial stiffness progression. Left: outcome 1 (increases in/persistently high baPWV). Right: outcome 2 (incident elevated baPWV). BaPWV indicates brachial-ankle pulse wave veloctiy; MAP, mean arterial pressure; TG, triglyceride; HDL-C, high-density lipoprotein cholesterol

We further performed ROC analyses to study the predictive power of baseline logTG and logTG/HDL-C (Fig. 3 and Table 2). When adding logTG or logTG/HDL-C to the basic model (age + MAP), the prediction of the outcomes showed an increase in AUC. For example, AUC for outcome 1 increased from 0.73 to 0.74 when logTG was added. Positive NRI and IDI indicated that adding logTG or logTG/HDL-C significantly improved risk reclassification for both outcomes.

**Table 2:** Model Performance for arterial stiffness progression

Models	AUC (95% CI)	NRI(95% CI)	p value	IDI (95% CI)	p value
*Outcome 1: Increase in/persistently high baPWV*					
Age + MAP	0.73 (0.70–0.77)	–	–	–	–
Age + MAP + LogTG	0.74 (0.71–0.78)	22.9% (7.8–37.9%)	0.003	1.1% (0.4–1.9%)	0.004
Age + MAP + LogTG/HDL-C	0.74 (0.71–0.78)	22.2% (7.0–37.4%)	0.004	1.2% (0.4–2.0%)	0.003
*Outcome 2: Incident elevated baPWV*					
Age + MAP	0.74 (0.69–0.79)	–	–	–	–
Age + MAP + LogTG	0.75 (0.70–0.81)	40.7% (18.8–62.6%)	< 0.001	1.4% (0.1–2.6%)	0.03
Age + MAP + LogTG/HDL-C	0.75 (0.70–0.80)	32.1% (10.1–54.1%)	0.004	1.1% (0.0–2.2%)	0.06

*AUC* area under the receiver operating curve, *NRI* net reclassification improvement, *IDI* integrated discrimination improvement, *MAP* mean arterial pressure, *TG* triglyceride, *HDL-C* high-density lipoprotein cholesterol

### Arterial stiffness progression and baseline lipid parameters as categorical variables

**Fig. 4:**
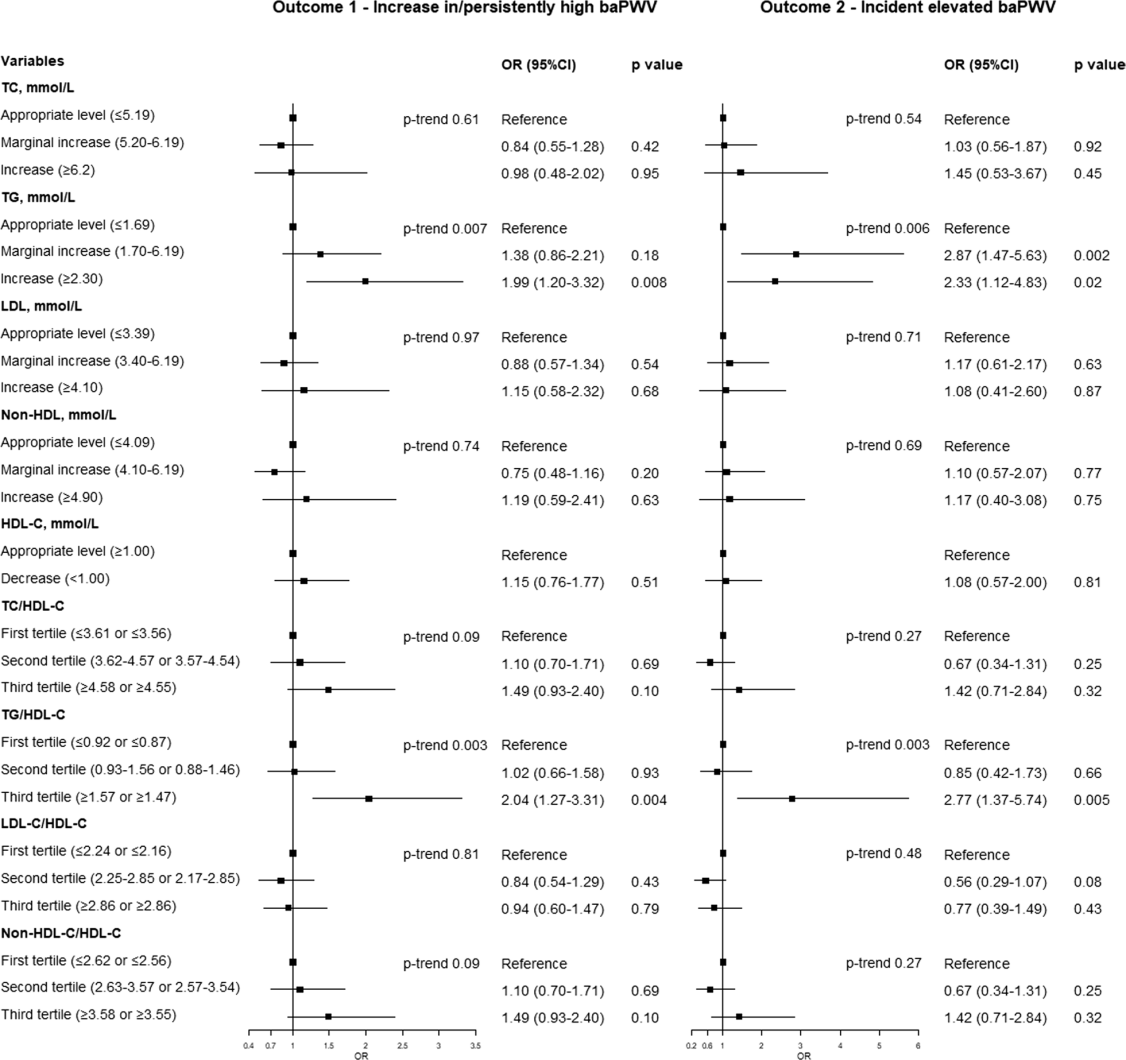
Forest plot of ORs of categorical lipids for arterial stiffness progrssion. Left: outcome 1 (increases in/persistently high baPWV). Right: outcome 2 (incident elevated baPWV). All ORs were adjusted for baseline age, mean arterial pressure, body mass index, smoking, heart rate, fasting blood glucose, creatinine, and uric acid. BaPWV indicates brachial-ankle pulse wave veloctiy; TC, total cholesterol; TG, triglyceride; LDL-C, low-density lipoprotein cholesterol; HDL-C, high-density lipoprotein cholesterol

In sensitivity analysis, serum lipids were converted to categorical variables (Fig. 4). TG and TG/HDL-C were significantly associated with both outcome 1 and outcome 2. The ORs associated with the increased versus appropriate level of TG (≥ 2.30 versus ≤ 1.69 mmol/L) was 1.99 (95% CI: 1.20–3.32) for outcome 1 and 2.33 (95% CI: 1.12–4.83) for outcome 2. The ORs associated with the highest versus lowest quartile of TG/HDL was 2.04 (95% CI: 1.27–3.31; ≥ 1.57 versus ≤ 0.92) for outcome 1 and 2.77 (95% CI: 1.37–5.74; ≥ 1.47 vs. ≤ 0.87) for outcome 2.

## Discussion

In this study, we compared the predictive power of baseline serum lipid parameters on arterial stiffness progression in a sample of apparently healthy Chinese men. We found that even after adjusting for the confounding factors, high baseline TG and TG/HDL-C were correlated with high risk of increases in/persistently high baPWV and incident elevated baPWV. In addition, we also found that serum TG was positively associated with baPWV in 794 healthy elderly subjects in a previous study [10]. Hence we confirmed a correlation between TG and arterial stiffness in mid- and late-life.

High TG is a traditional risk factor for CVD [21]. However, previous investigations, which have included TG in multivariable regression analyses for arterial stiffness progression, were conflicting. Some studies showed that baseline TG levels and changes of TG levels over time independently predicted arterial stiffness progression after adjusting for other cardiovascular risk factors [22–25], while others have not [26–29]. TG/HDL-C has been considered as an independent predictor of insulin resistance [30, 31], diabetes mellitus [32], and CVD [33]. High-level TG/HDL-C ratio was also proved to be associated with higher arterial stiffness in cross-sectional studies [34–36], and the relationship might be non-linear [34]. TG/HDL-C was also found to be an independent determinant of arterial stiffness in adolescents and young adults [37]. Consistent with our study, subjects with high TG/HDL-C ratio also had high risk of carotid-femoral pulse wave velocity (cfPWV) progression in healthy individuals [38]. However, the result of another investigation revealed that higher cfPWV in late-life was not related to faster annual rates of change in TG/HDL-C from mid-life [36]. The conflicting results of these investigations were likely based on the differences in the study populations and the evaluation indexes of arterial stiffness and we provided evidence of the independent predictive value of baseline TG and TG/HDL for baPWV progression in Chinese healthy males.

It has long been thought that arterial stiffness is only a feature of hypertension-mediated organ damage, while more recent studies show that arterial stiffness precedes hypertension [39, 40]. Arterial stiffness reflects the cumulative damage of cardiovascular risk factors on the vascular wall and is even better than the blood pressure in reflecting the risk of CVD [41]. Arterial stiffness begins in early childhood and shows increasing progression in adults [7]. Our study population displayed a mean baPWV increase of 16 cm/s per year. The progression rate was quite similar to that of another group of Chinese males, in which the 5-year change was 70 cm/s for the healthy men [23]. The average annual change of baPWV of a Japanese/American population-based cohort study was 9 cm/s, but this study merely focused on men aged 40 to 49 years [42]. Valid baPWV progression rates have not been established so far.

China is the world’s largest consumer of tobacco, and more than a third of the included individual in our study were current smokers at baseline, which was consistent with the finding of national surveys done in China [43]. There is evidence indicating that cigarette smoking can alter the critical enzymes of lipid transport and therefore exerts negative effects on the lipid and lipoprotein profile and [44]. Smoking increases levels of TC, TG, and LDL-C, while decreases the cardio-protective HDL-C [44]. Meanwhile, previous longitudinal studies have demonstrated that continuous smoking accelerated the arterial stiffness progression in both adolescents and adults even at low levels of smoking exposure [29, 45]. It may be meaningful to study the mediating effects of lipids in the association between smoking and vascular elasticity damage, but there has been very little research into this subject yet. Moreover, detailed data on both time since quitting among past smokers and pack-years among current smokers were not available in the present study, and we did not find a significant relationship between smoking status as a dichotomous yes/no measure and arterial stiffness progression. But we showed that baseline TG and TG/HDL-C were able to predict arterial stiffness progression even after simultaneous adjustment for smoking and other risk factors. Interestingly, studies on the role of lipids in the association between smoking and CVD have been published but far from a convincing conclusion [46–48].

We have some other limitations to consider. First, although the results were adjusted for multiple covariates that may be associated with baPWV, the possibility of residual confounds remains. Second, we referred to some articles about cfPWV, because data regarding baPWV progression is limited. Although baPWV is highly correlated with cfPWV, we do not think the results are interchangeable. Third, the results of this study may not be generalizable to women and individuals with comorbidities. Moreover, whether controlling for TG and HDL-C can attenuate progression of arterial stiffness or whether any long-term reduction in arterial stiffness can translate into a reduction in cardiovascular events has not been directly demonstrated in randomized controlled trials.

## Conclusions

In conclusion, we provided evidence in this longitudinal study that baseline TG and TG/HDL-C which could be obtained from routine serum lipids independently predicted increases in/persistently high baPWV and incident elevated baPWV, and they were superior to other traditional lipid variables in identifying healthy men who are at an increased risk of arterial stiffness progression. This positive association between TG, TG/HDL-C, and baPWV progression should be considered in the management of vascular health.

## Data Availability

The datasets analyzed in the present study are available from the corresponding author on reasonable request.

## Abbreviations

CVD: Cardiovascular disease
BaPWV: Brachial-ankle pulse wave velocity
TC: Total cholesterol
TG: Triglyceride
LDL-C: Low-density lipoprotein cholesterol
HDL-C: High-density lipoprotein cholesterol
SBP: Systolic blood pressure
DBP: Diastolic blood pressure
FBG: Fasting blood glucose
ABI: Ankle-brachial index
BMI: Body mass index
MAP: Mean arterial pressure
OR: Odds ratio
CI: Confidence interval
ROC: Receiver operating characteristic
AUC: Area under the receiver operating characteristic
NRI: Net reclassification improvement
IDI: Integrated discrimination improvement
CfPWV: Carotid-femoral pulse wave velocity
